# TyG index is associated with microalbuminuria in a large population-based study: independent and exploratory interaction analyses

**DOI:** 10.1371/journal.pone.0355437

**Published:** 2026-08-05

**Authors:** Xiaomin Lin, Xudong Huang

**Affiliations:** Department of Clinical Laboratory, Jieyang People’s Hospital, Jieyang, Guangdong, China; Sri Ramachandra Institute of Higher Education and Research (Deemed to be University), INDIA

## Abstract

**Background:**

Insulin resistance surrogates are associated with microalbuminuria; however, population-scale evidence for the triglyceride–glucose (TyG) index remains scarce. We examined the cross-sectional association between TyG index and microalbuminuria in a large Chinese cohort and explored whether diabetes modifies this link.

**Methods:**

We analyzed 33,416 adults from eight regional centers. Microalbuminuria was defined as urinary albumin-to-creatinine ratio ≥ 30 mg/g. Multivariate logistic regression and restricted cubic splines were used to estimate odds ratios (ORs) per 1-unit TyG increase and the potential nonlinear relationship between TyG index and microalbuminuria risk. The effects of diabetes and other factors were tested using interaction terms.

**Results:**

The mean age was 57.7 ± 9.3 years, and the microalbuminuria prevalence was 14.5%. TyG index was independently associated with microalbuminuria after adjustment for age, sex, body mass index, waist, hip circumference, blood pressure, lipids, liver function indices, sleep variables, estimated glomerular filtration rate, smoking, alcohol, and diabetes (OR, 1.28; 95% confidence interval [CI], 1.20–1.36; *P* < 0.001). Restricted cubic spline analysis demonstrated a linear association between TyG index and the odds of microalbuminuria (*P* for overall association < 0.001; *P* for non-linearity = 0.187). The association was present in participants with (OR, 1.46; 95% CI, 1.32–1.60) and without diabetes (OR, 1.13; 95% CI, 1.04–1.23). The interaction with diabetes status was significant (*P* < 0.001; false discovery rate-corrected *P* = 0.008), indicating a stronger association in patients with diabetes. The sensitivity analyses confirmed the robustness of the main findings.

**Conclusions:**

The TyG index was linearly associated with microalbuminuria. This association was observed in patients with and without diabetes, with a stronger estimated effect among those with diabetes. TyG may serve as a simple, readily available marker for identifying individuals with higher odds of microalbuminuria across glycemic status; however, longitudinal studies are needed to establish temporal relationships.

## Introduction

Chronic kidney disease (CKD) is estimated to affect approximately 10% of the population, or 800 million individuals globally [1]. Microalbuminuria, as reflected by the urinary albumin-to-creatinine ratio (UACR), is recognized as an early marker of kidney injury [2–4]. Additionally, microalbuminuria often indicates vascular damage and is closely associated with cardiovascular complications of various diseases [5–8]. Given the important role of microalbuminuria in CKD and cardiovascular disease and the large and growing burden of cardiovascular disease and CKD worldwide, timely identification of individuals at high risk for microalbuminuria is critical in both the prevention and management of CKD and cardiovascular disease. Therefore, the early identification of individuals with increased albumin excretion remains a public health priority [9].

The triglyceride–glucose (TyG) index, a simple ln-based composite of fasting triglycerides and glucose, has emerged as a reliable proxy for insulin resistance (IR) [10,11]. IR is a pathological condition characterized by diminished cellular response to insulin, leading to metabolic dysregulation that contributes to the pathogenesis of several chronic diseases [12]. Because insulin receptors are expressed in the vasculature and kidneys, in addition to classical insulin-targeting tissues, IR may influence the renal regulation of glucose uptake, glomerular function regulation, gluconeogenesis, and tubular transport [13]. According to previous studies, IR and/or its contributing variables may be harmful at the onset of CKD [14]. A recent study has suggested that the effect of IR on mortality in patients with albuminuric diabetic nephropathy may be mediated by its association with albuminuria [15]. Prior studies support a positive association between the TyG index and microalbuminuria as well as renal injury; however, these studies have predominantly focused on specific chronic disease populations, with relatively small sample sizes [16–18]. Evidence in the general population without diabetes remains sparse, and the potential difference in the association between TyG and microalbuminuria among individuals with and without diabetes remains unclear. Notably, the few general-population studies that included individuals without diabetes have generally reported null associations in that subgroup, leaving it unclear whether the TyG–microalbuminuria association extends beyond diabetes. The present large multicenter analysis was designed to address this gap.

Therefore, we analyzed 33,416 adults from a nationwide multicenter population-based survey to quantify the cross-sectional association between the TyG index and microalbuminuria. Considering that the degree of IR varies in different populations, such as those with poor lifestyle, environmental, and psychological factors in the population without diabetes, this may lead to an increased susceptibility to microalbuminuria, which may affect the results of our study. Accordingly, we specifically focused on whether this association was consistent between individuals with and without diabetes.

## Methods

### Study design and data sources

This cross-sectional study aimed to evaluate the relationship between the TyG index and microalbuminuria and to explore whether diabetes modifies this association by stratifying the population with and without diabetes.

This was a secondary analysis of a publicly available dataset from Ye et al. [19], which was not specified in the original study protocol. The original study by Ye et al. investigated the associations of self-reported sleep duration and daytime napping with renal hyperfiltration and microalbuminuria, which was also an outcome in that report. The present study addresses an entirely different exposure—the TyG index—which was not examined in the original publication, and we additionally adjusted for the sleep variables identified in the parent study. Accordingly, no overlap exists between the exposure–outcome associations reported here and those in the original article, and these findings should be considered exploratory and hypothesis-generating. Data were obtained from the China Multicenter Longitudinal Study of Diabetes Cancer Risk Assessment. After applying the exclusion criteria (primary kidney disease, ACEI/ARB medication use, and extreme sleep duration), the study cohort comprised 33,850 participants from eight regional Chinese centers. The original study was approved by the Institutional Review Board of Ruijin Hospital, Shanghai Jiao Tong University, and all the participants provided written informed consent. The authors had no access to any identifying information during or after the analysis, therefore, additional ethical approval was not required for this retrospective analysis.

### Missing value handling

**Fig 1:**
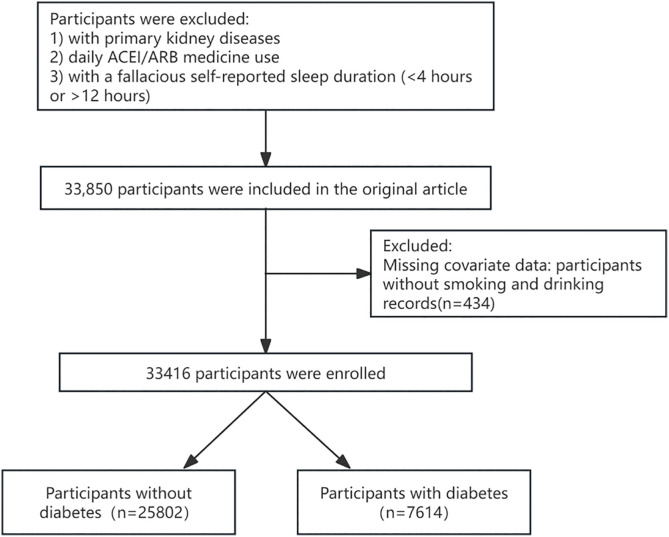
Process flow diagram for the study’s population inclusion.

Missing data were handled using complete-case analysis (listwise deletion) because the proportion of missing data was low (< 2% for all variables). This approach is unlikely to have introduced material bias given the low level of missingness; therefore, multiple imputations were not undertaken. Building on the original study, we excluded 434 individuals with incomplete covariate information, primarily those with missing smoking or alcohol consumption data. Ultimately, 33,416 participants were included in the study. The participant selection procedure is illustrated in Fig 1.

### Clinical and laboratory measurements

In the initial study, basic demographic information and laboratory data were collected by trained medical staff through standardized surveys. Demographic information included age, sex, smoking status, alcohol consumption, and family history of diabetes. Participants’ height, weight, hip circumference (HC), waist circumference (WC), diastolic blood pressure (DBP), and systolic blood pressure (SBP) were measured. Blood samples were collected early in the morning after an 8-hour fast the night before for biochemical tests, including triglyceride (TG), total cholesterol (TC), low-density lipoprotein cholesterol (LDL), high-density lipoprotein cholesterol (HDL), creatinine (Scr), serum urea nitrogen (BUN), alanine aminotransferase (ALT), aspartate aminotransferase (AST), gamma–glutamyl transpeptidase (GGT), fasting blood glucose (FBG), glycated hemoglobin (HbA1c), fasting blood insulin, and postprandial blood insulin. Participants without a history of diabetes underwent a 75 g oral glucose tolerance test, whereas those with diabetes underwent a 100 g oral glucose tolerance test, after which a venous blood sample was drawn for postprandial blood glucose measurement at 120 min.

### UACR measurement

UACR was measured after a urine sample was collected in the morning. Microalbuminuria was defined as a UACR ≥ 30 mg/g, which was calculated as follows: UACR = urine albumin (mg/dl) / urine creatinine (g/dl).

### Calculation and assessment of the TyG index

The formula below was used to determine the TyG index:

$\text{TyG\textasciitilde{}index}=\ln\left[\frac{\text{fasting\textasciitilde{}triglycerides\textasciitilde{}(mg/dL)}\times \text{fasting\textasciitilde{}glucose\textasciitilde{}(mg/dL)}}{2}\right]\text{.}$

### Definitions

FBG ≥ 7.0 mmol/L, PBG ≥ 11.1 mmol/L, or a documented medical history of diabetes were the criteria for diabetes mellitus. An average systolic blood pressure ≥ 130 mm Hg or a diastolic blood pressure ≥ 80 mm Hg after three measurements, or a history of hypertension, are considered indicators of hypertension.

The estimated glomerular filtration rate (eGFR) was calculated using the Modification of Diet in Renal Disease formula. CKD was defined as an eGFR < 60 ml/min, which was calculated as follows: eGFR(ml/min * 1.73 m^2^) = 186 * Scr(mg/dl)^−1.154^ * age(years)^-0.203^(female * 0.742)

### Statistical analysis and assessment of covariates

Continuous variables are presented as means ± standard deviations or as medians; categorical variables are described as percentages. TyG was analyzed per 1-unit increase and across quartiles (Q1 = reference). Quartiles were selected a priori because they yielded four equally sized groups, allowing a test for a dose–response trend without imposing a specific functional form, limiting the influence of extreme values, and were consistent with the categorization most commonly used in prior TyG–kidney studies, facilitating comparison. Logistic regression supplied odds ratios (ORs) and 95% confidence intervals (CIs) for microalbuminuria.

The relationship between TyG index and microalbuminuria was analyzed using univariate and multivariate binary logistic regression analyses. Models with and without multivariate adjustment were used. The variance inflation factor (VIF) was used to test for probable multicollinearity; a VIF ≥ 5 indicates multicollinearity. Because the TyG index incorporates fasting glucose, fasting glucose, and HbA1c, which were deliberately not entered as covariates to avoid overadjustment and collinearity, diabetes status—an a priori confounder and our pre-specified effect modifier—was retained and modelled both without (model III) and with (model IV) adjustment, complemented by diabetes-stratified analyses. After covariate screening by calculating VIF values (S1 Table), clinical expertise, and relevant literature on covariates, four incremental models were built. Model I was adjusted for age and sex. Model II was adjusted for SBP, DBP, WC, HC, BMI, alcohol consumption, and smoking status, based on Model 1. Model III was further adjusted for biochemical markers (ALT, AST, GGT, eGFR, HDL, LDL, total sleep duration, and daytime napping) in all the participants. Model IV was fully adjusted for the diabetes status based on Model III. In the diabetes-stratified analyses, diabetes status could not serve as a covariate; therefore, sleep variables were added at the final modelling step (Model IV). In addition to analyzing the TyG index as a continuous variable, we transformed the TyG index into a categorical variable based on quartiles, using the lowest quartile group (quartile 1) as a reference, calculated the p-value of the trend to assess the dose-response relationship, and validated the results obtained with the continuous TyG index. Diagnostic procedures (posterior predictive analysis, binned residual analysis, leverage–residual plot, multicollinearity test, and model fit indices) were used to check the key model assumptions.

We used a restricted cubic spline (RCS) model to generate smoothed curves and investigate the potential nonlinear relationship between the TyG index and risk of microalbuminuria. Nonlinearity was assessed by comparing the model with only linear terms and the model containing both linear and cubic terms. If a nonlinear relationship was identified, the threshold effects were examined.

Sensitivity analyses were performed to ensure the robustness and reliability of the findings. Subgroup analyses were conducted via multivariate logistic regression to evaluate heterogeneity between subgroups (sex, age ≤ 60 vs. > 60 years, smoking status, alcohol consumption status, obesity status, or hypertension), with each stratum fully adjusted for covariates other than the stratification variables. A likelihood ratio test (*P* < 0.05) was used to assess the interactions between the TyG index and subgroup variables on the risk of microalbuminuria. Multiple testing correction was performed using the Benjamini–Hochberg method to control the false discovery rate (FDR), with an FDR-corrected *P* < 0.05 considered statistically significant. We conducted an additive interaction assessment for the subgroups with statistically significant interaction effects. The additive interactions were evaluated using the relative excess risk of interaction (RERI), and its statistical significance was evaluated using 95% CI. For this analysis, the TyG index was dichotomized based on quartiles: Q1–Q2 were defined as low TyG, and Q3–Q4 as high TyG. The RERI quantifies the excess risk of microalbuminuria attributable to the interaction between a high TyG index and diabetes status beyond the sum of their individual effects. In addition, E-values were computed to evaluate the possible influence of unmeasured confounders on our findings.

In our study, we did not precalculate the statistical power because the sample size was based entirely on existing data. The R software (version 4.2.2, http://www.R-project.org) and a Free Statistical analysis platform (version 2.2, Beijing Free Clinical Medical Technology Co., Ltd.) were used for all the studies. All analyses were performed with a two-sided *P* < 0.05, indicating a statistically significant difference.

## Results

### Baseline characteristics of the participants

The study included 33,416 participants with a mean age of 57.7 ± 9.3 years, of whom 11,081 (33.2%) were male, and 22,335 (66.8%) were female. In addition, 77.2% had no diabetes, and 22.8% had diabetes (Table 1). The TyG index was normally distributed with a mean value of 8.8. The median UACR was 10 mg/g. The prevalence of microalbuminuria was 14.5%. The participants were grouped according to TyG index quartiles, which revealed a progressive increase in the proportion of patients with microalbuminuria, CKD, diabetes, and hypertension from Q1 to Q4.

**Table 1:** Baseline traits of the participants categorized by the TyG index.

Variables	Total	Q1(6.6–8.4)	Q2(8.4–8.7)	Q3(8.7–9.2)	Q4(9.2–12.3)	*P* value
Participants (numbers)	33416	8354	8352	8356	8354	
Age (years)	57.7 ± 9.3	55.6 ± 9.4	57.7 ± 9.2	58.5 ± 9.2	58.9 ± 9.0	< 0.001
Sex (%)						< 0.001
Male	11081 (33.2)	2499 (29.9)	2641 (31.6)	2754 (33.0)	3187 (38.1)	
Female	22335 (66.8)	5855 (70.1)	5711 (68.4)	5602 (67.0)	5167 (61.9)	
Smoking (%)						< 0.001
No	28354 (84.9)	7258 (86.9)	7150 (85.6)	7150 (85.6)	6796 (81.4)	
Yes	5062 (15.1)	1096 (13.1)	1202 (14.4)	1206 (14.4)	1558 (18.6)	
Drinking (%)						0.068
No	24717 (74.0)	6189 (74.1)	6138 (73.5)	6264 (75.0)	6126 (73.3)	
Yes	8699 (26.0)	2165 (25.9)	2214 (26.5)	2092 (25.0)	2228 (26.7)	
WC (cm)	86.2 ± 10.0	82.0 ± 9.7	85.1 ± 9.7	87.7 ± 9.6	90.0 ± 9.2	< 0.001
HC (cm)	97.0 ± 7.8	94.5 ± 7.6	96.4 ± 7.6	98.1 ± 7.7	99.1 ± 7.6	< 0.001
Scr (mmol/L)	68.6 ± 17.0	65.3 ± 14.8	67.6 ± 16.3	69.3 ± 17.5	72.1 ± 18.5	< 0.001
HDL (mmol/L)	1.3 ± 0.3	1.5 ± 0.4	1.4 ± 0.3	1.3 ± 0.3	1.2 ± 0.3	< 0.001
LDL (mmol/L)	3.0 ± 0.9	2.7 ± 0.8	3.0 ± 0.9	3.2 ± 0.9	3.0 ± 0.9	< 0.001
TC (mmol/L)	5.1 ± 1.1	4.6 ± 1.1	5.0 ± 1.1	5.2 ± 1.1	5.4 ± 1.2	< 0.001
TG (mmol/L)	1.4 (1.0, 2.0)	0.8 (0.7, 0.9)	1.2 (1.1, 1.3)	1.7 (1.5, 1.9)	2.6 (2.2, 3.4)	< 0.001
ALT (mmol/L)	15.0 (11.0, 21.0)	13.0 (9.0, 17.0)	14.0 (10.0, 19.0)	15.0 (11.0, 22.0)	18.0 (13.0, 26.0)	< 0.001
AST (mmol/L)	22.4 ± 12.5	20.9 ± 11.9	21.9 ± 11.0	22.8 ± 11.8	24.2 ± 14.8	< 0.001
GGT (mmol/L)	21.0 (15.0, 32.0)	16.0 (12.0, 23.0)	19.0 (14.0, 28.0)	22.0 (16.0, 33.0)	28.0 (20.0, 44.0)	< 0.001
FBG (mmol/L)	6.0 ± 1.7	5.2 ± 0.6	5.6 ± 0.9	5.9 ± 1.2	7.2 ± 2.6	< 0.001
PBG (mmol/L)	8.5 ± 4.0	6.7 ± 2.2	7.6 ± 2.8	8.7 ± 3.5	11.2 ± 5.2	< 0.001
HbA1c (%)	6.1 ± 1.1	5.7 ± 0.5	5.9 ± 0.6	6.1 ± 0.8	6.7 ± 1.6	< 0.001
BMI (kg/m^2^)	24.7 ± 3.7	23.3 ± 3.5	24.3 ± 3.6	25.2 ± 3.6	25.9 ± 3.5	< 0.001
eGFR (ml/(min × 1.73m2)	93.9 ± 19.1	99.1 ± 20.9	94.5 ± 17.9	92.2 ± 17.6	89.9 ± 18.4	< 0.001
SBP (mmHg)	131.9 ± 20.4	125.8 ± 19.2	130.6 ± 19.7	133.9 ± 20.4	137.4 ± 20.4	< 0.001
DBP (mmHg)	77.6 ± 10.8	74.6 ± 10.6	76.9 ± 10.6	78.5 ± 10.7	80.4 ± 10.7	< 0.001
Tumor (%)						0.226
Yes	979(2.9)	234 (2.8)	232 (2.8)	241 (2.9)	272 (3.3)	
No	32437 (97.1)	8120 (97.2)	8120 (97.2)	8115 (97.1)	8082 (96.7)	
Obesity (%)						< 0.001
No	28338 (84.8)	7734 (92.6)	7348 (88.0)	6809 (81.5)	6447 (77.2)	
Yes	5078 (15.2)	620 (7.4)	1004 (12.0)	1547 (18.5)	1907 (22.8)	
Diabetes (%)						< 0.001
No	25802 (77.2)	7839 (93.8)	7251 (86.8)	6359 (76.1)	4353 (52.1)	
Yes	7614 (22.8)	515 (6.2)	1101 (13.2)	1997 (23.9)	4001 (47.9)	
CKD (%)						< 0.001
No	32597 (97.5)	8247 (98.7)	8199 (98.2)	8154 (97.6)	7997 (95.7)	
Yes	819(2.5)	107 (1.3)	153 (1.8)	202 (2.4)	357 (4.3)	
Hypertension, n (%)						< 0.001
No	13240 (39.6)	4574 (54.8)	3568 (42.7)	2891 (34.6)	2207 (26.4)	
Yes	20176 (60.4)	3780 (45.2)	4784 (57.3)	5465 (65.4)	6147 (73.6)	
UACR (mg/g)	10.0 (5.8, 20.0)	8.4 (5.1, 15.7)	9.6 (5.7, 18.5)	10.2 (6.0, 20.4)	12.7 (6.7, 25.9)	< 0.001
Microalbuminuria (%)						< 0.001
No	28558 (85.5)	7509 (89.9)	7302 (87.4)	7135 (85.4)	6612 (79.1)	
Yes	4858 (14.5)	845 (10.1)	1050 (12.6)	1221 (14.6)	1742 (20.9)	
TyG index	8.8 ± 0.6	8.1 ± 0.2	8.6 ± 0.1	8.9 ± 0.1	9.6 ± 0.4	< 0.001
Total sleep duration (hours)	8.1 ± 1.2	8.0 ± 1.2	8.1 ± 1.2	8.1 ± 1.2	8.2 ± 1.2	< 0.001
Daytime napping (%)						< 0.001
No	22149 (66.3)	5949 (71.2)	5625 (67.3)	5392 (64.5)	5183 (62.0)	
Yes	11267 (33.7)	2405 (28.8)	2727 (32.7)	2964 (35.5)	3171 (38.0)	

Continuous variables are expressed as the mean ± SD or median (interquartile ranges), whereas categorical variables are expressed as n (%).

### Associations of the TyG index with microalbuminuria risk in all participants

Univariate logistic regression analysis (S2 Table) demonstrated that various factors significantly impacted the risk of microalbuminuria. For instance, HbA1c, TyG index, age, BMI, WC, HC, TG, AST, ALT, GGT, SBP, DBP, FBG, PBG, and total sleep duration were positively correlated with the risk of microalbuminuria (*P* < 0.001). In contrast, TC, LDL, and HDL levels negatively correlated with the risk of microalbuminuria. Furthermore, being female or having obesity, hypertension, or diabetes increased the risk of microalbuminuria. In addition, non-smoking and non-drinking were associated with a higher rate of microalbuminuria than smoking and drinking. The probability of microalbuminuria increased by 72% for every unit rise in the TyG index (OR, 1.72; 95% CI, 1.64–1.80; *P* < 0.001).

After performing a one-way logistic regression analysis, we constructed four multifactorial logistic regression models to assess the relationship between the TyG index and the risk of microalbuminuria (Table 2). Model I OR 1.67 (95% CI, 1.59–1.76; *P* < 0.001); Model II OR 1.56 (95% CI, 1.48–1.64; *P* < 0.001); Model III (without diabetes adjustment) OR 1.44 (95% CI, 1.36–1.52; *P* < 0.001) and fully adjusted Model IV OR 1.28 (95% CI, 1.20–1.36; *P* < 0.001).

**Table 2:** Association of the TyG index with microalbuminuria in all participants.

Variable	Crude.OR (95%CI)	*P* value	Model I (OR, 95%CI)	*P* value	Model II (OR, 95%CI)	*P* value	Model III (OR, 95%CI)	*P* value	Model Ⅳ (OR, 95%CI)	*P* value
TyG index	1.72 (1.64 ~ 1.8)	<0.001	1.67 (1.59 ~ 1.76)	<0.001	1.56 (1.48 ~ 1.64)	<0.001	1.44 (1.36 ~ 1.52)	<0.001	1.28 (1.2 ~ 1.36)	<0.001
TyG index quartile										
Q1	1(Ref)		1(Ref)		1(Ref)		1(Ref)		1(Ref)	
Q2	1.28 (1.16 ~ 1.41)	<0.001	1.18 (1.07 ~ 1.30)	0.001	1.12 (1.02 ~ 1.24)	0.021	1.12 (1.02 ~ 1.24)	0.024	1.08 (0.98 ~ 1.2)	0.116
Q3	1.52 (1.38 ~ 1.67)	<0.001	1.36 (1.24 ~ 1.5)	<0.001	1.24 (1.13 ~ 1.37)	<0.001	1.2 (1.09 ~ 1.33)	<0.001	1.1 (0.99 ~ 1.22)	0.065
Q4	2.34 (2.14 ~ 2.56)	<0.001	2.12 (1.94 ~ 2.32)	<0.001	1.85 (1.69 ~ 2.04)	<0.001	1.63 (1.47 ~ 1.81)	<0.001	1.35 (1.21 ~ 1.51)	<0.001
*P* for trend		<0.001		<0.001		<0.001		<0.001		<0.001

Model I: Adjusted for age and sex.

Model II: model 1 plus BMI, waist, hip circumference, SBP, DBP, smoking, and alcohol.

Model III: model 2 plus HDL, LDL, ALT, AST, GGT, eGFR, and sleep variables (total sleep duration and daytime napping).

Model IV: model 3 plus diabetes status.

OR, odds ratio; CI, confidence interval.

In addition, we assessed the relationship between the TyG index quartiles and the risk of microalbuminuria in all models. The risk of microalbuminuria increased progressively with increasing TyG index quartiles (*P* for trend < 0.001). These findings emphasize the robustness of the positive association between the TyG index and risk of microalbuminuria, even after extensive adjustment for potential confounders.

**Fig 2:**
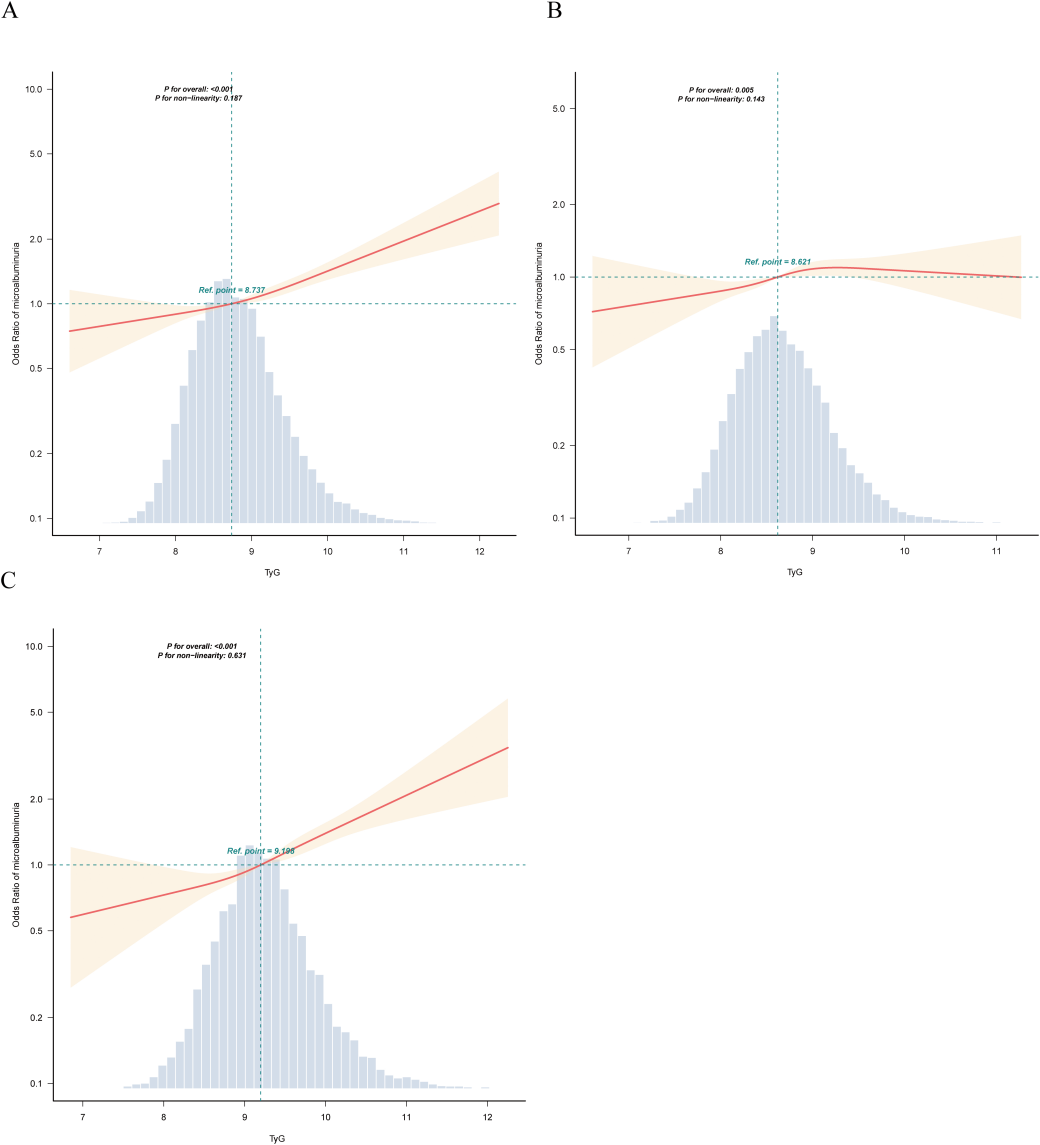
Smoothed curve fitting of the TyG index and risk of microalbuminuria for all participants (A), participants without diabetes (B), and participants with diabetes (C). Panel A was adjusted for age, sex, BMI, WC, HC, SBP, DBP, smoking, alcohol, HDL, LDL, ALT, AST, GGT, eGFR, diabetes status, total sleep duration and daytime napping. Panel B and Panel C were adjusted for age, sex, BMI, WC, HC, SBP, DBP, smoking, alcohol, HDL, LDL, ALT, AST, GGT, eGFR, total sleep duration and daytime napping.The red line indicates the estimated relationship, and the orange area indicates the 95% confidence interval.

As shown in Fig 2A, we used the RCS to visualize the association between the TyG index and risk of microalbuminuria in all participants. The risk of microalbuminuria in all participants was positively and linearly correlated with the TyG score after all factors were considered in Model IV of Table 2 (*P* < 0.001, *P* for nonlinearity  =  0.187).

### TyG index and microalbuminuria by diabetes status

As shown in Tables 3 and 4, in participants without diabetes, each one-unit increase in TyG was associated with 13% higher odds of microalbuminuria after full adjustment (OR, 1.13; 95% CI, 1.04–1.23; *P* < 0.001); the corresponding estimate in those with diabetes was 46% (OR, 1.46; 95% CI, 1.32–1.60; *P* < 0.001). Across the TyG quartiles, a graded increase in odds was observed in both groups (*P* for trend < 0.001). The results of the curve-fitting analysis (Figs 2B and 2C) showed a positive linear correlation between the TyG index and the risk of microalbuminuria, both in the populations with and without diabetes, after adjusting for all covariates in Model IV in Table 3 (*P* < 0.05, *P* for nonlinearity > 0.05), with no evidence of a threshold effect.

**Table 3:** Association between TyG and the risk for microalbuminuria in participants without diabetes.

Variable	Crude.OR (95%CI)	*P* value	Model I (OR, 95%CI)	*P* value	Model II (OR, 95%CI)	*P* value	Model III (OR, 95%CI)	*P* value	Model Ⅳ (OR, 95%CI)	*P* value
TyG index	1.39 (1.3 ~ 1.49)	<0.001	1.34 (1.25 ~ 1.44)	<0.001	1.25 (1.16 ~ 1.34)	<0.001	1.16 (1.07 ~ 1.25)	<0.001	1.13 (1.04 ~ 1.23)	0.003
TyG quartile										
Q1	1(Ref)		1(Ref)		1(Ref)		1(Ref)		1(Ref)	
Q2	1.23 (1.1 ~ 1.38)	<0.001	1.15 (1.02 ~ 1.29)	0.02	1.1 (0.98 ~ 1.23)	0.115	1.14 (1.02 ~ 1.29)	0.027	1.12 (1 ~ 1.26)	0.059
Q3	1.4 (1.25 ~ 1.56)	<0.001	1.26 (1.12 ~ 1.41)	<0.001	1.17 (1.04 ~ 1.31)	0.008	1.23 (1.09 ~ 1.39)	0.001	1.19 (1.05 ~ 1.34)	0.006
Q4	1.69 (1.52 ~ 1.88)	<0.001	1.55 (1.39 ~ 1.73)	<0.001	1.38 (1.23 ~ 1.55)	<0.001	1.32 (1.16 ~ 1.5)	<0.001	1.27 (1.12 ~ 1.44)	<0.001
*P* for trend		<0.001		<0.001		<0.001		<0.001		<0.001

Model I: Adjusted for age and sex.

Model II: model 1 plus BMI, waist, hip circumference, SBP, DBP, smoking, and alcohol.

Model III: model 2 plus HDL, LDL, ALT, AST, GGT, and eGFR.

Model IV(Fully Adjusted): model 3 plus sleep variables (total sleep duration and daytime napping).

OR, odds ratio; CI, confidence interval.

**Table 4:** Association between the TyG index and risk for microalbuminuria in participants with diabetes.

Variable	crude.OR (95%CI)	*P* value	Model I (OR, 95%CI)	*P* value	Model II (OR, 95%CI)	*P* value	Model III (OR, 95%CI)	*P* value	Model Ⅳ (OR, 95%CI)	*P* value
TyG index	1.56 (1.44 ~ 1.7)	<0.001	1.64 (1.51 ~ 1.78)	<0.001	1.59 (1.45 ~ 1.73)	<0.001	1.46 (1.33 ~ 1.6)	<0.001	1.46 (1.32 ~ 1.6)	<0.001
TyG index quartile										
Q1	1(Ref)		1(Ref)		1(Ref)		1(Ref)		1(Ref)	
Q2	1.29 (1.1 ~ 1.52)	0.002	1.28 (1.08 ~ 1.5)	0.004	1.23 (1.04 ~ 1.45)	0.017	1.19 (1.01 ~ 1.41)	0.043	1.18 (1 ~ 1.4)	0.056
Q3	1.67 (1.42 ~ 1.95)	<0.001	1.66 (1.42 ~ 1.95)	<0.001	1.59 (1.35 ~ 1.87)	<0.001	1.52 (1.28 ~ 1.8)	<0.001	1.49 (1.25 ~ 1.77)	0.001
Q4	2.05 (1.76 ~ 2.4)	<0.001	2.16 (1.85 ~ 2.53)	<0.001	2.02 (1.72 ~ 2.38)	<0.001	1.77 (1.48 ~ 2.1)	<0.001	1.74 (1.46 ~ 2.08)	<0.001
*P* for trend		<0.001		<0.001		<0.001		<0.001		<0.001

Model I: Adjusted for age and sex.

Model II: model 1 plus BMI, waist, hip circumference, SBP, DBP, smoking, and alcohol.

Model III: model 2 plus HDL, LDL, ALT, AST, GGT, and eGFR.

Model IV(Fully Adjusted): model 3 plus sleep variables (total sleep duration and daytime napping).

OR, odds ratio; CI, confidence interval.

### Subgroup and sensitivity analyses

**Fig 3:**
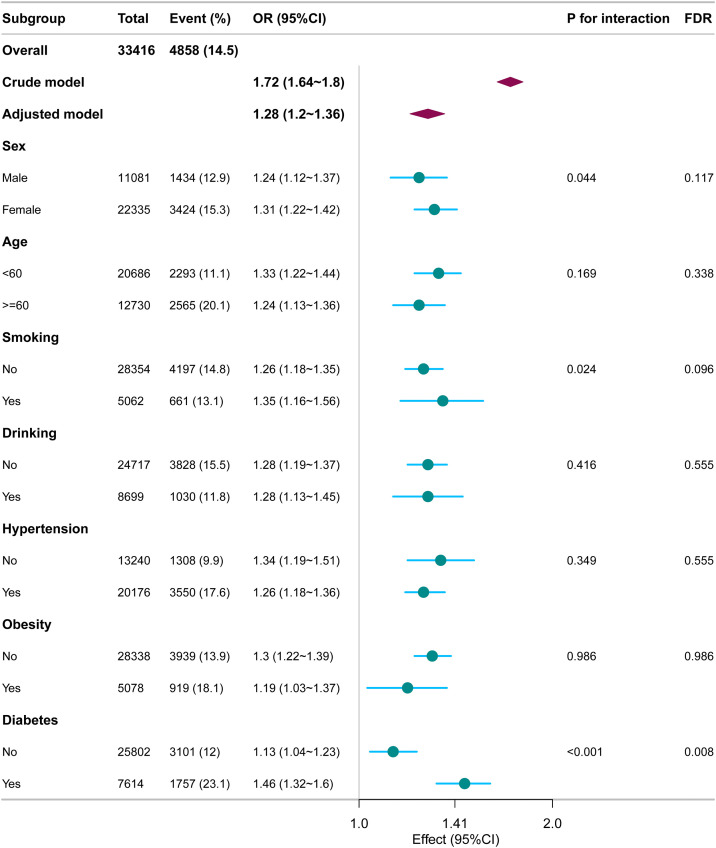
Subgroup analysis of the relationship between TyG index and microalbuminuria risk in all participants. The models were adjusted for age, sex, BMI, waist, hip circumference, SBP, DBP, smoking, alcohol, HDL, LDL, ALT, AST, GGT, eGFR, diabetes status, total sleep duration and daytime napping, if not stratified. The *P* values for the interaction effect were determined via the likelihood ratio test. OR, odds ratio. CI, confidence interval.

As shown in Fig 3, we further evaluated, through subgroup analyses in all participants, the OR effect of the TyG index on the microalbuminuria risk across subgroups defined by sex, age (<60 vs. ≥ 60 years), smoking, alcohol consumption, diabetes, obesity, and hypertension, as well as the interactions between these variables. The TyG index was significantly associated with microalbuminuria in all the subgroups examined. The association was quantitatively stronger in individuals with diabetes than in those without diabetes (OR, 1.46; 95% CI, 1.32–1.60 vs. OR, 1.13; 95% CI, 1.04–1.23; *P* for interaction < 0.001; FDR-corrected *P* = 0.008), with both multiplicative (OR, 1.30; 95% CI, 1.10–1.55) and additive (RERI = 0.47, 95% CI, 0.24–0.70) interactions reaching statistical significance. No other subgroup interactions survived multiple testing corrections (all FDR-corrected, *P* > 0.05). In both populations with and without diabetes, no interactions with any other factors were detected in the respective subgroup analyses (all *P* for interaction > 0.05) (S1 Fig).

We systematically evaluated the key model assumptions using diagnostic procedures (S2 Fig). The posterior predictive check showed satisfactory overall model fit; binned residual analysis indicated that the linearity assumption was mostly satisfied, with only minor deviations noted at lower predicted probabilities; the leverage–residual plot revealed no influential observations; and the VIF confirmed that all predictors were below the threshold of 5, indicating no multicollinearity issues. Additionally, we performed sensitivity analyses using E-value tests to assess the effects of unmeasured confounders. The E-values were 1.88 (total population), 1.51 (population without diabetes) and 2.28 (population with diabetes), which were higher than the minimum combined strength of unmeasured confounding factors required to eliminate the exposure-outcome association to a null effect (S3 Fig). Therefore, unmeasured confounding factors may be unlikely to influence our findings in either population.

## Discussion

In this cross-sectional study of 33,416 Chinese adults, a 1-unit increase in the TyG index was linearly associated with 28% higher odds of microalbuminuria after multivariate adjustment. The association was present in both participants with (OR, 1.46; 95% CI, 1.32–1.60) and without diabetes (OR, 1.13; 95% CI, 1.04–1.23) participants, with a significantly stronger effect in individuals with diabetes (*P* for interaction < 0.001). Both multiplicative and additive interactions were statistically significant. No statistically significant interactions were observed among other subgroups. Restricted cubic spline analysis demonstrated a linear dose–response relationship across the entire observed TyG range in the overall population (*P* for overall association < 0.001; *P* for non-linearity = 0.187), with no evidence of a threshold effect. Similar linear patterns were observed in both subgroups with (*P* for overall association < 0.001; *P* for non-linearity = 0.631) and without diabetes (*P* for overall association = 0.005; *P* for non-linearity = 0.143) subgroups. The model assumption checks and E-value analyses supported the robustness of our findings.

Previous studies have demonstrated a positive association between the TyG index and the risk of microalbuminuria; however, research has predominantly focused on populations with specific chronic diseases. Patients with diabetes represent the most extensively studied population, with a significant positive correlation consistently observed in this group [16,20–22]. Few studies have examined the general population, and although these studies have shown a positive association between TyG and microalbuminuria, subgroup analyses in individuals without diabetes have yielded non-significant results. For instance, an analysis of National Health and Nutrition Examination Survey data has reported a significant positive association between TyG and albuminuria in U.S. adults (OR, 1.37; 95% CI, 1.15–1.63). No significant association was detected in U.S. adults without diabetes (OR, 0.98; 95% CI, 0.77–1.23), with no significant interaction by diabetes status (*P* = 0.41) [23]. Similarly, the Northern Shanghai Study has revealed no significant association between TyG levels and microalbuminuria in 2,180 older individuals with diabetes [17]. By contrast, after conducting stratified regression and sensitivity analyses in a large general population cohort, we observed that TyG was significantly associated with microalbuminuria in individuals with (OR, 1.46; 95% CI, 1.32–1.60) and without diabetes (OR, 1.13; 95% CI, 1.04–1.23), with a significant interaction by diabetes status (*P* < 0.001). This quantitative difference may reflect the fact that patients with diabetes often exhibit severe IR, as well as poor lifestyle and environmental and psychological factors that may increase susceptibility to microalbuminuria, amplifying the effect of TyG in this population. To the best of our knowledge, this is the first study to stratify a large general population cohort by diabetes status and conduct in-depth analyses of the association between TyG and microalbuminuria in both subgroups with and without diabetes. Our findings extend prior reports by demonstrating that the TyG–microalbuminuria association is not confined to populations with diabetes, but exists across glycemic statuses, albeit with quantitative differences. The discrepancy between our results on individuals without diabetes and those of previous studies likely reflects our substantially larger sample size, broader age range, and geographical representation.

The TyG index is a simple, low-cost surrogate for IR that can be easily implemented in routine practice [24,25]. Under physiological conditions, IR affects numerous organs and insulin regulatory pathways. Insulin receptors are extensively expressed in classical insulin-responsive organs such as the liver, skeletal muscle, and white adipose tissue, in addition to peripheral nontraditional insulin target tissues such as the kidney. In the kidney, insulin receptors are expressed in the glomerular cells, podocytes, renal tubular cells, and other cells [13]. Therefore, IR can directly affect the kidney (especially by impairing the insulin signaling pathway of the renal tubules and glomeruli, leading to glomerular renal tubular dysfunction and podocyte injury, thereby affecting the overall filtration and excretion functions of the kidney) and may indirectly affect the occurrence and development of renal function through lipid abnormalities and oxidative stress responses [13]. The expansion of adipose tissue leads to an increase in leptin production, and the pro-inflammatory effect of the hepatokine fetuin-A may lead to proteinuria [26,27].

The strengths of this study include a large multicenter sample, detailed subgroup analyses, and robustness to unmeasured confounding factors. However, this study has some limitations. First, this was a cross-sectional study; therefore, the observed association between TyG index and microalbuminuria does not imply causality or a temporal sequence. As this analysis was not prespecified, these findings should be considered exploratory. Longitudinal studies are warranted to determine whether elevated TyG levels precede microalbuminuria. Second, microalbuminuria was defined by a single-spot UACR measurement without repeat confirmation. Additionally, information on the exclusion of transient causes (e.g., urinary tract infection, fever, and strenuous exercise) and specific assay standardization across centers was not available in the original dataset, which may have affected the diagnostic accuracy. Third, despite a comprehensive adjustment for demographic, metabolic, and lifestyle factors, residual confounding from unmeasured or imprecisely measured variables (e.g., dietary patterns, physical activity intensity, genetic predisposition, and medication adherence) cannot be fully excluded. Fourth, our sample comprised Chinese adults from eight regional centers, and the generalizability of these findings to other ethnicities or populations with different metabolic profiles requires further investigation. Finally, we did not evaluate insulin-based indices, such as HOMA-IR. The TyG index was selected specifically because unlike HOMA-IR, it does not require insulin assays and is therefore more accessible in routine clinical practice, although a direct comparison with HOMA-IR in this cohort would be informative and warrants future studies.

## Conclusion

In this large cross-sectional study, the TyG index was linearly associated with microalbuminuria. This association was observed in adults with and without diabetes, with a stronger estimated effect in those with diabetes. TyG may serve as a simple, readily available marker for identifying individuals at a higher risk of microalbuminuria across various glycemic statuses. However, as this is a cross-sectional study, causality cannot be inferred, and future cohort studies are needed to establish the temporal relationship and causal association between the TyG index and microalbuminuria.

## Supporting information

S1 Table Variance inflation factor (VIF) for independent variables. (XLSX)

S2 Table Univariate logistic regression analysis of factors associated with microalbuminuria. (XLS)

S1 Fig Subgroup analyses for the relationship between the TyG index and the risk of microalbuminuria in participants without diabetes (A) and participants with diabetes (B). Models were adjusted for age, sex, BMI, waist circumference, hip circumference, SBP, DBP, smoking, alcohol, HDL, LDL, ALT, AST, GGT, eGFR, total sleep duration, and daytime napping, when not stratified. The *P* values for the interaction effect were determined via the likelihood ratio test. OR, odds ratio; CI, confidence interval.
(TIF)

S2 Fig Model Diagnostics: (A) Posterior Predictive Check; (B) Binned Residuals Analysis; (C) Influential Observations (Leverage vs. Standardized Residuals); (D) Collinearity Assessment (Variance Inflation Factor, VIF); (E) Model Fit Indices. (TIF)

S3 Fig Sensitivity analysis of E-values for the association between the TyG index and risk for microalbuminuria in all participants (A), participants without diabetes (B) and participants with diabetes (C). (TIF)
